# Magnitude and associated factors of postpartum family planning uptake among postpartum women in Ethiopia: an umbrella review

**DOI:** 10.3389/fgwh.2024.1481601

**Published:** 2024-12-18

**Authors:** Teketel Ermias Geltore, Simegn Alemu, Abiy Tadesse Angelo, Teketel Tesfaye Mamito, Workneh Elias Orsongo, Lakew Lafebo Foto, Tesfahun Simon Hadaro

**Affiliations:** ^1^ Department of Midwifery, College of Medicine and Health Science, Wachemo University Durame Campus, Durame, Ethiopia; ^2^ Department of Nursing, Mizan Tepi University, Mizan Aman, Ethiopia; ^3^ Department of Emergency and Critical Nursing, College of Medicine and Health Science, Wolaita Sodo University, Sodo, Ethiopia; ^4^ School of Public Health, Institute of Health Science, Bule Hora University, Bule Hora, Ethiopia; ^5^ Department of Midwifery, College of Medicine and Health Science, Ariba Minch University, Ariba Minch, Ethiopia

**Keywords:** postpartum, family planning, SR and MA, utilization, Ethiopia

## Abstract

**Background:**

The World Health Organization indicates that despite advancements, the rates of maternal and neonatal mortality and morbidity during the postpartum period continue to be alarmingly high. Furthermore, untapped opportunities to enhance maternal health and promote effective newborn care, including family planning services, have not been fully leveraged. Earlier meta-analyses and systematic reviews have addressed this subject; however, a thorough evidence synthesis has not been provided. Therefore, the objective of this study was to compile the existing systematic reviews (SRs) concerning postpartum family planning uptake among postpartum women in Ethiopia.

**Method:**

This review used an umbrella review method, incorporating numerous systematic reviews. We followed the Preferred Reporting Items for Systematic Reviews and Meta-Analyses (PRISMA) guidelines and the Meta-analysis of Observational Studies guideline (MOOSE). A comprehensive literature review was conducted across prominent four electronic databases (including MEDLINE/PubMed, Cochrane, Web of Science and Science Direct) from June 15, to July 15, 2024. This review encompassed investigations carried out within the uptake of family planning among post-partum women and its determinants in Ethiopia were the primary outcome. A set of inclusion criteria was established to identify all pertinent systematic reviews, including studies, with no restrictions on data collection and publication year. The quality of the methods was evaluated using the Assessment of Multiple Systematic Reviews tool, (AMSTAR) tool. Statistical analysis was conducted using STATA version 17 software, and the 95% confidence interval was utilized to establish statistical significance. I-squared statistics were employed to evaluate the heterogeneity of the studies by using a random-effects meta-analysis model.

**Results:**

The umbrella review includes five studies with a total of 44,276 postpartum women. The pooled prevalence of postpartum family planning utilization was 36.41% (95% CI: 24.78, 48.03). Family planning counseling (AOR: 4.12, 95% CI: 2.89, 4.71), couple discussion (AOR: 3.06, 95% CI: 1.42, 5.60), and postnatal follow-up (AOR: 3.48, 95% CI: 2.60, 4.83) were significantly associated with postpartum family planning uptake.

**Conclusion:**

The study results indicate that the adoption of postpartum family planning in Ethiopia requires focused intervention. This can be achieved by identifying and enhancing community frameworks to involve men in reproductive health initiatives and by providing comprehensive family planning information and services during postnatal care. Addressing the aforementioned factors is crucial to mitigate the risks associated with unintended pregnancies and to manage the swift increase in population.

**Systematic Review Registration:**

https://www.crd.york.ac.uk/prospero/display_record.php?ID=CRD42024568435, PROSPERO (CRD42024568435).

## Introduction

The postnatal period, lasting up to six weeks after birth, is crucial for women, newborns, and families. Despite this, maternal and neonatal mortality rates remain high, and opportunities to enhance maternal health and newborn care are often missed (1). Woman should wait at least two years between pregnancies to ensure proper care for the most recent child and reduce the risk of maternal and child mortality (2, 3). Family planning can save lives, but there is a lack of understanding about postpartum fertility and birth control among educators, healthcare providers, and the users (4, 5).

To foster a more promising future for everyone, Sustainable Development Goal 3 aims to lower the maternal mortality ratio to below 70 per 100,000 live births by the year 2030. This goal includes the commitment to provide universal access to sexual and reproductive health care services, encompassing family planning (5). More than 90% of women worldwide want to delay or avoid pregnancy within a year of giving birth. In sub Saharan Africa, this number increases to 95%, but about 70% of women in these areas do not use contraception (6).

Unplanned pregnancies within a year after childbirth can arise from various factors. Majorly, ovulation can resume as early as 25 days post-delivery, and about 23% of women have a sexual activity before the six-week mark, if there is no contraception (7). Whether pregnancies are intended or unintended, every woman should be able to use contraception during the postpartum period if she chooses, in order to promote her own and her family's health (2).

If a woman becomes pregnant as early as she giving birth, then it can lead to low birth weight, a doubling of the chances of premature birth, and a 60% increase in the risk of infant mortality for babies born less than 24 months after a previous birth (8). Review of literature showed that marital status, secondary and above level of education, maternal age, longer birth interval after delivery, ever used contraceptive methods, menses resumption, starting sex, antenatal care follow-up, postnatal care, knowledge about family planning, and discussion with husband (4, 9–13) were found to be determinants of postpartum contraceptive utilization.

Research in Ethiopia shows that 47% of pregnancies occur within 24 months of the previous birth (14), having the highest maternal mortality rate in Sub-Saharan Africa at 412 per 100,000 live births (15). Furthermore, there is a high demand for postpartum family planning (PPFP), with rates as high as 86% within the first 5 months after childbirth, decreasing to 76% within the first year. Additionally, 81% of women do not use PPFP because they are unaware that they can conceive within a year after giving birth (16). To improve postpartum contraceptive use, provision of health education, counseling about the importance of FP, access to various family planning methods are paramount factors (17).

There are significant differences in Ethiopia regarding awareness and use of modern contraceptives. Nearly all married women in Addis Ababa know at least one contraceptive method, compared to only 67% in the Somali region. Urban women have a higher adoption rate (48%) than rural women (38%). The public sector provides 87% of modern contraceptives, while the private sector offers 12%. Usage rates are highest in Addis Ababa (48%) and Amhara (50%), and lowest in Somali (3%) and Afar (13%) (18, 19). This data suggested that there are no standard approaches to helping Ethiopian women who wish to utilize family planning per their needs. The 2016 EDHS data shows that 25% of women use modern family planning six months postpartum, with significant differences based on delivery location: 18% for home births and 43% for facility births (20). These disparities may result from variations in access to healthcare and demographic factors influencing childbirth location and contraceptive use. The report's inconsistencies hinder clinical decision-making. Despite previous studies and reviews (21–25), policymakers and providers still lack a comprehensive overview of the evidence on factors influencing postpartum family planning demand. This study aims to present a comprehensive overview of systematic reviews (SRs) to consolidate the current evidence concerning the uptake of modern postpartum family planning (PPFP) and contributing factors among women women in the postpartum periods.

## Methods

### Study design, data source and search strategy

**Figure 1:**
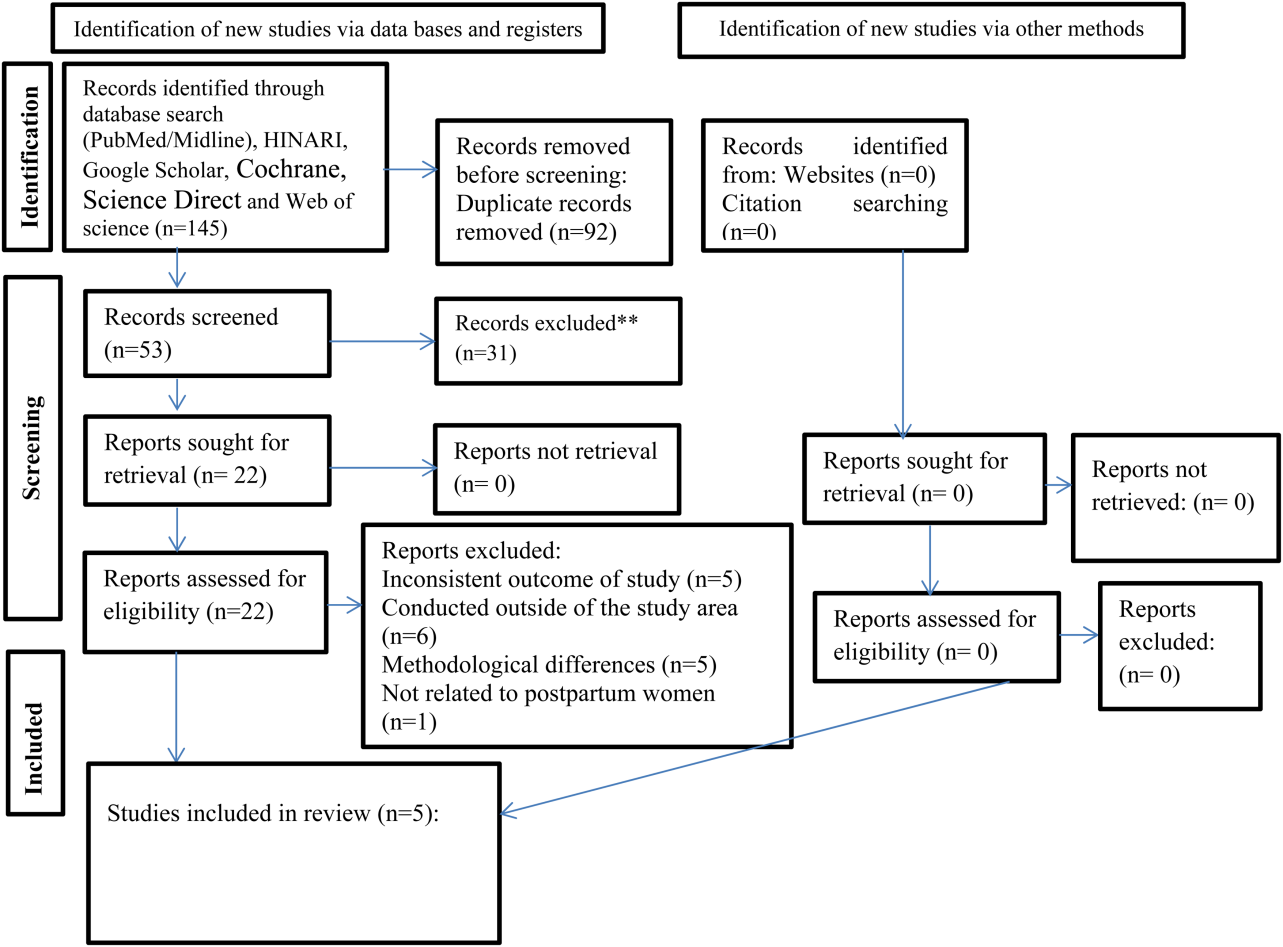
PRISMA flow diagram of studies included in the final umbrella review of the magnitude of PPFP uptake in Ethiopia.

A preliminary search was carried out in PROSPERO to examine the current research landscape and mitigate the duplication risk. At that point, no analogous studies were discovered. Subsequently, a research protocol was formulated and registered in PROSPERO (CRD42024568435). The search terms were integrated using Boolean operators “OR” and “AND”. The following MeSH terms or keywords were applied in the online database: postpartum OR post-delivery OR parturition OR puerperium OR immediate postpartum OR extended postpartum AND prevalence OR magnitude OR proportion AND use OR utilization OR intention OR unmet need OR barrier AND predictors OR contraception OR contraceptive OR family planning OR modern contraceptives OR modern postpartum family planning OR modern family planning AND Ethiopia AND systematic review (Supplementary Table S1). The Meta-analysis of Observational Studies guideline (MOOSE) (26, 27). These methodologies include detailed checklists with 35 elements to guide the execution and documentation of observational studies at high risk of bias and confounding, particularly in evaluating retrospective data. The systematic reviews (SRs) and meta-analyses concerning the adoption of modern contraceptive postpartum family planning (PPFP) per the PRISMA guidelines (28) was used (Figure 1). An extensive literature review was performed utilizing four major electronic databases, such as MEDLINE/PubMed, Cochrane, Web of Science and Science Direct covering the period from June 15, 2024 to July 15, 2024.

### Eligibility criteria

A set of inclusion and exclusion criteria was established to identify all pertinent systematic reviews: (i) population: studies focusing on postpartum mothers, (ii) outcome: adoption of modern postpartum family planning (PPFP) and its determining factors, (iii) language: all published studies in English, (iv) study design: systematic reviews and meta-analyses, (v) geographical area: research conducted exclusively in Ethiopia. The exclusion criteria for reviews were determined based on the following factors: the presence of similar duplicate reviews published across multiple journals, articles that did not report the intended outcome (i.e., incomplete quantitative data), and reviews that failed to present a clear research question, search strategy, or a defined methodology for article selection.

### Outcome measurement, study selection, and quality assessment

#### PICOS guide

The PICOS framework highlights four critical components: population, intervention, comparison, and outcome. Its primary aim is to identify and evaluate the clinical aspects of evidence throughout the systematic review process. The PICOS components in this study were delineated as follows: Population: postpartum women in Ethiopia; Intervention: postpartum family planning; Comparison: No adoption of modern PPFP; Outcomes: overall prevalence of modern contraceptive uptake among postpartum women and the factors associated with it in Ethiopia; Study design: systematic reviews or meta-analyses. Each study in the analysis underwent a thorough evaluation using the AMSTAR tool, which consists of 11 questions to assess methodological and evidential integrity. Quality was rated on a scale of 0–11, with scores indicating high 8–10, medium 4–7, or low quality <3 (29).

#### Data extraction process

Date from included studies was extracted using standardized extraction tool developed in an Excel spreadsheet and labeled as follows. For each SRM, the following information was extracted: (1) Identification data (first author's last name and publication year), (2) Review aim, (3) Prevalence or proportion of uptake of postpartum family planning, (4) Risk factors, (5) Odds ratio or relative risk with 95% confidence intervals for the risk factors, (6) Number of primary studies included within each SRM study and their respective design type, (7) Total number of sample size included, (8) Publication bias assessment methods and scores, (9) Quality assessment methods and scores, (10) Data synthesis methods (random or fixed-effects model), and (11) The authors' main conclusion of the SRM study (12). TEG and SA independently extracted information on study characteristics and key findings from each review as stated above. In cases of disagreement, additional input was sought from a third and fourth author LLF and ATA, respectively. The study did not employ a specific search strategy, and there were no limitations regarding publication years. Records were organized using Endnote version 8.

#### Data synthesis and statistical analysis

Data synthesis in the included SRM studies involved both qualitative and quantitative methods. Multiple estimates for modern PPFP prevalence and associated factors were presented as a range, with an aggregated estimate calculated using STATA version 17. Study heterogeneity was assessed using *I*^2^ and Cochran Q statistics (30). A random effects model with a 95% CI was used to determine the pooled prevalence of modern PPFP utilization. Due to the inadequate number of studies incorporated in this umbrella review, we did not appraise publication bias. To successfully assess the publication bias, at least 10 studies is required (31). Stata version 17.0 software was used for analyses.

### Ethical consideration

In this research, it was unnecessary to obtain consent or ethical approval from the participants, as the study utilized data derived from SRM studies.

## Results

### Study selection

The included studies that fulfilled the specified criteria are presented in Figure 1. A total of 145 articles were initially gathered from four distinct databases. PubMed/Midline generated 58 research articles, Cochrane generated 41 articles, Science Direct generated 12, and Web of science Identified 34 records through reference list review (34). Subsequently, after eliminating 92 duplicate entries, we were left with 53 records. A review of the titles and abstracts led to the exclusion of 31 articles. The remaining 22 articles were then assessed for eligibility. Ultimately, 17 articles were excluded for several reasons; five were inconsistent with the outcome of study, six were conducted outside of the study area, five had a methodological differences and one was not related to postpartum women. In the end, 5 studies (21–25) were found to meet the eligibility requirements for inclusion. This umbrella review encompasses five systematic review and meta-analysis (21–25), which were derived from observational primary studies. These primary studies consisted of 3 cohort studies, 2 case-control studies, and 77 cross sectional studies, amounting to a total of 82 studies. The collective sample size across these studies involved 44,276 postpartum but one study included women of reproductive age group, pregnant and post-partum (23) The number of primary studies varied per SRM, ranging from 12 (23) to 19 (24). Additionally, the sample size per meta-analysis exhibited variability, spanning from 4,367 (23) to 11,932 (24). Two SRM studies were published in the year 2020 (24, 25) and one each were published in 2021 (23), 2022 (22) and 2023 (21). These studies comprehensively examined both the prevalence and determinants of postpartum family planning uptake. Based on the included SRMA the prevalence of postpartum family planning were ranged from 21.04, (13.08, 29.00), *I*^2^ = 98.43% (21) to 48.11 (36.96, 59.27), *I*^2^ = 99.4 (25). These statistics highlight the diversity in prevalence rates across the studies. General characteristics of the systematic review and meta-analyses studies are presented (Table 1).

**Table 1:** Characteristic of the systematic reviews of modern contraceptives utilization and its associated factors among postpartum women in Ethiopia, 2024.

Authors (year)	Review objective	Search strategy	Reported prevalence	Included studies	Population	Risk of bias	Sample size	AMSTAR quality
Silesh et al. (2023) (22)	To assess the pooled estimate of immediate postpartum family planning utilization and its associated factors in Ethiopia	Electronic databases were used to conduct an extensive search of all published studies, and the digital library was used to identify any unpublished studies. Search date was until July 30, 2022. Clear searching terms were defined. All available studies from 2016 to 2020 were included. Clear inclusion and exclusion criteria's were defined.	21.04% (95% CI:13.08, 29.00), *I*^2^ = 98.43%	15 (13 cross sectional and 2 case control)	Postpartum women	The quality of included studies were appraised clearly using NOS	6,444	10
A. Tesfu et al. (2022) (23)	To estimate the pooled prevalence and factors associated with postpartum modern contraceptive use in Ethiopia.	PubMed, MEDLINE, EMBASE, Hinari, Google Scholar, direct Google search, African Journal Online (AJOL), online repository, and gray pieces of literature were used. Search date was before April 30, 2019. Clear searching terms were defined. Studies conducted in Ethiopia from 2012 to 2019 were included. Clear inclusion and exclusion criteria's were defined	45.44% (95% CI: 31.47, 59.42), *I*^2^ = 99.7%	18 (16 cross sectional and 2 cohort)	Postpartum mothers	The quality of the studies has been transparently assessed	10,883	10
Kassa et al. (2021) (24)	To assess post-partum intrauterine contraceptive device utilization and its associated factors among women in Ethiopia	PubMed, Google Scholar, EMBASE, HINAR, Scopus, Web of Sciences, and Grey literature. The searching periods were from January 12, 2020, to February 12, 2020. Clear searching terms was defined. Clear inclusion and exclusion criteria were defined. All available studies from 2015 to 2021 were included	21.63% (95% CI: 14.46, 28.81), *I*^2^ = 99.7%	12 (all were cross sectional)	Women of reproductive age group, pregnant and post-partum women	The quality of included studies were appraised clearly	4,367	9
Wakuma et al. (2020) (25)	To determine the best available pieces of evidence to pool the magnitude of postpartum modern contraception utilization and find out its determinants	Medline, Scopes, PubMed, CINAHL, Embase, Cochrane library, the JBI Library, the Web of Science, Google Scholar, and Google search engines. The search was conducted from November 14th to December 5th, 2019 without limiting the publication dates of the articles. Clear searching terms was defined. Clear inclusion and exclusion criteria were defined.	45.79% (95% CI: 36.45, 55.13). *I*^2^ = 99.2%	19 (18 were cross sectional and 1 cohort)	Postpartum women	The quality of included studies were appraised clearly using JBI-MAStARI	11,932	9
Tsegaye Mehare et al. (2020) (26)	To estimate the pooled prevalence of postpartum contraceptive use and determinants in Ethiopia using the accessible studies	MEDLINE through PubMed, Science Direct, EMBASE, HINARI, and Cochrane Library. The searching was carried out from the 13th of April to the 23rd of May 2019. All papers published in English from 2015 to May 23rd of 2019 were included in the review. From Google and Google Scholar, unpublished studies have been retrieved. Clear searching terms was defined. Clear inclusion and exclusion criteria were defined.	48.11% (95% CI: 36.96, 59.27), *I*^2^ = 99.4%	18 (all were cross sectional)	Postpartum women	The quality of included studies were appraised clearly using NOS	10,650	9

SR, systematic review; MA, meta-analysis; AMSTAR, assessment of multiple systematic reviews tool; NOS, Newcastle–Ottawa scale; JBI-MAStARI, The Joanna Briggs Institute Meta-Analysis of Statistics Assessment and Review Instrument.

### Methodological quality of the included SRM studies

Using the AMSTAR tool the methodological quality of included SRM studies evaluated. The quality of scoring was done out of 11 points and ranged from 9 to 10 (Table 2).

**Table 2:** Methodological quality of the included SRM studies based on AMSTAR tool in Ethiopia, 2024.

AMSTAR tool	Authors				
	Silesh et al.	A. Tesfu et al.	Kassa et al.	Wakuma et al.	Tsegaye Mehare et al.
Priori design provided	No	No	No	No	No
Duplicate study selection and data extraction	Yes	Yes	Yes	Yes	Yes
Search comprehensiveness	Yes	Yes	Yes	Yes	Yes
Inclusion of grey literature	Yes	Yes	Yes	Yes	Yes
Included and excluded studies provided	Yes	Yes	No	No	No
Characteristics of the included studies provided	Yes	Yes	Yes	Yes	Yes
Scientific quality of the primary studies assessed and documented	Yes	Yes	Yes	Yes	Yes
Scientific quality of included studies used appropriate conclusions	Yes	Yes	Yes	Yes	Yes
Appropriateness of methods used to combine studies’ findings	Yes	Yes	Yes	Yes	Yes
Conflict of interest	Yes	Yes	Yes	Yes	Yes
Likelihood of publication bias was assessed	Yes	Yes	Yes	Yes	Yes
Total (11)	10	10	9	9	9

### Pooled prevalence of modern postpartum family planning uptake in Ethiopia

**Figure 2:**
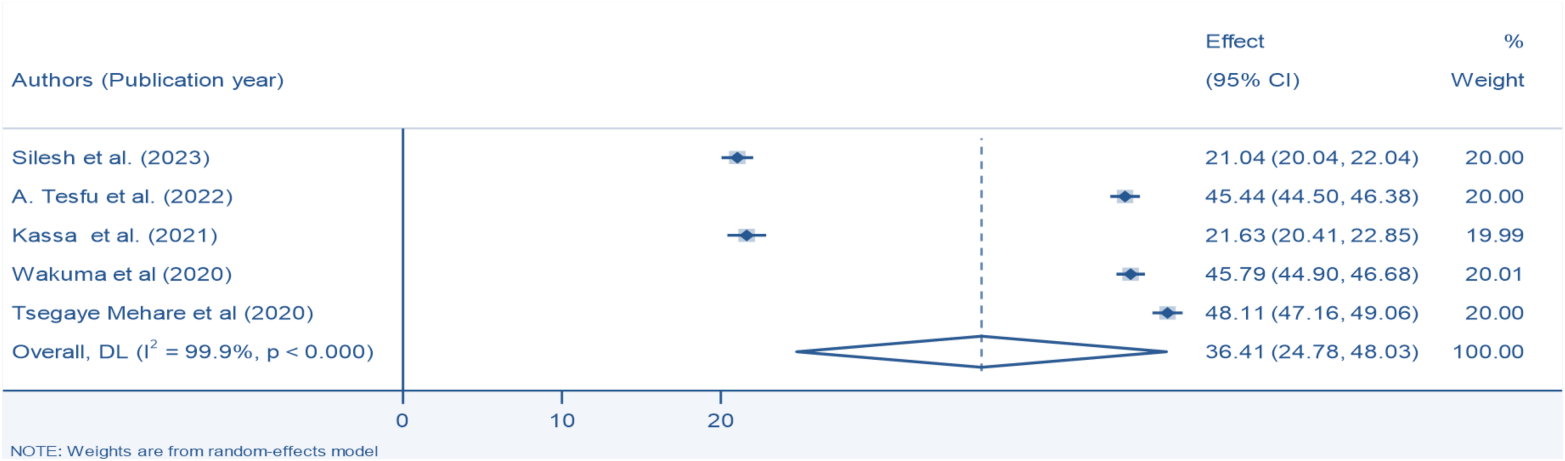
Pooled prevalence of modern postpartum family planning among postpartum women in Ethiopia, 2024.

From umbrella review of five SRM studies the pooled prevalence of PPFP utilization was 36.41% (95% CI, 24.78, 48.03) with the heterogeneity index (*I*^2^ = 99.9%, *P* < 0.000) showing substantial heterogeneity. Therefore, we have used the random effect model to resolve the issue of heterogeneity among the included reviews Figure 2.

### Subgroup analysis

**Figure 3:**
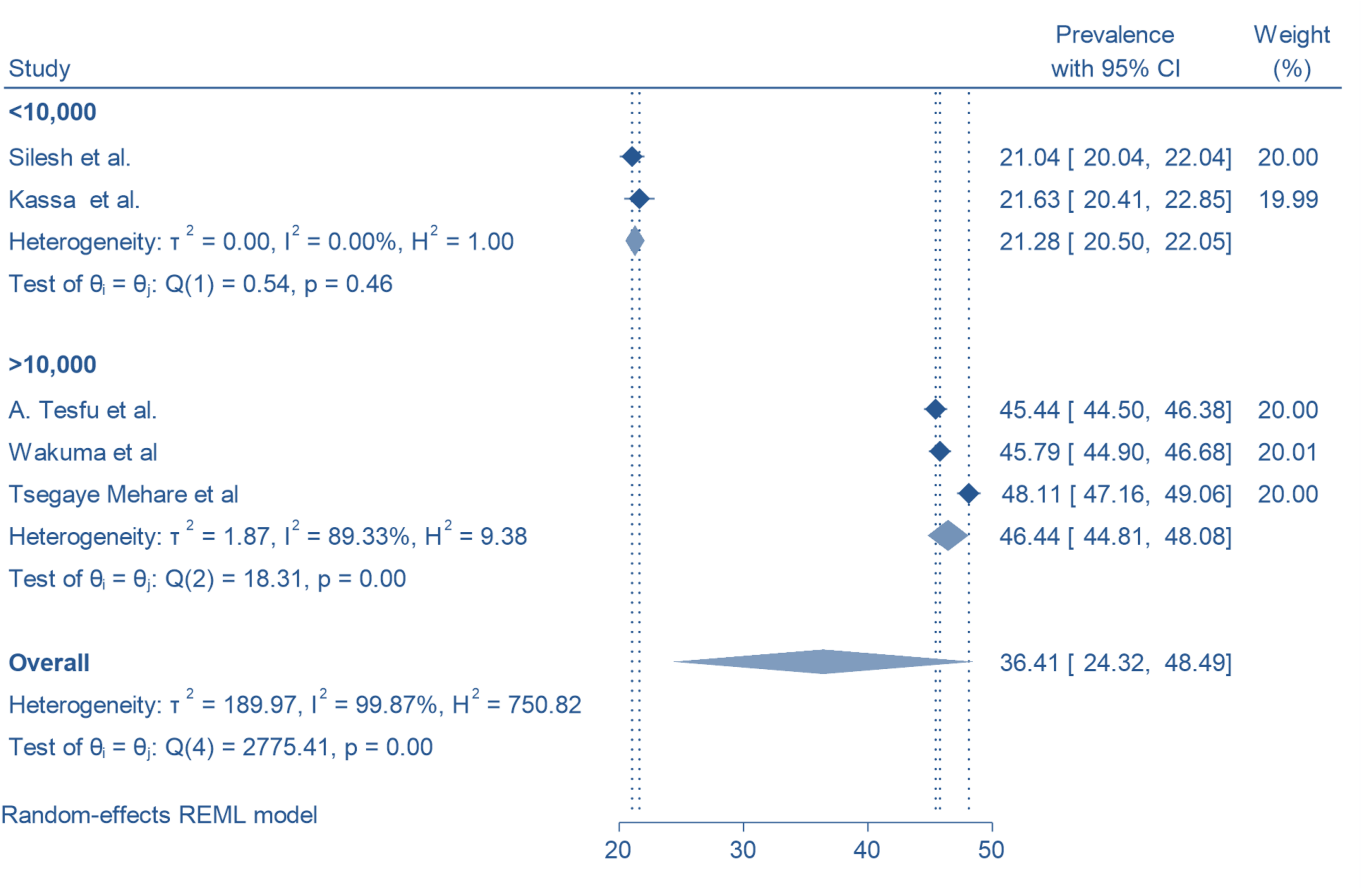
Subgroup analysis of the pooled prevalence of modern postpartum family planning among postpartum women in Ethiopia, 2024 by sample size.

The subgroup analysis focusing on sample size revealed that the sample greater than 10,000 had the highest prevalence of use of postpartum family planning 46.44% (95% CI, 44.81, 48.08), whereas sample size less than 10,000 had the lowest prevalence 21.28% (95% CI, 20.50, 22.05) Figure 3.

### Sensitivity analysis

**Figure 4:**
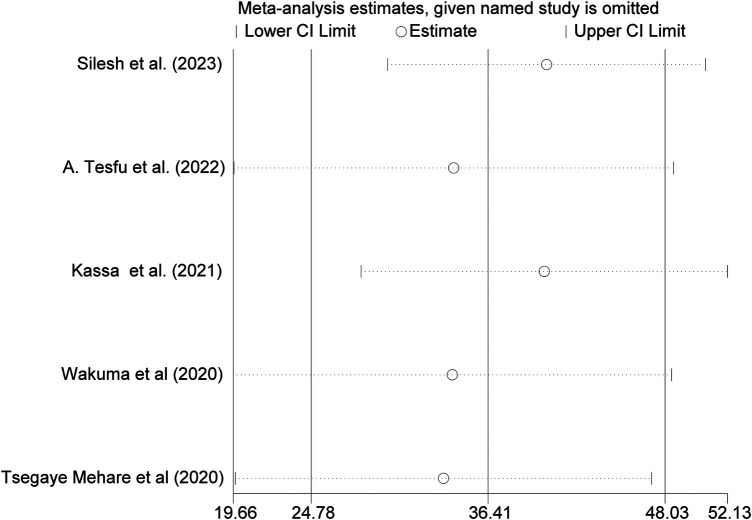
Sensitivity analyzes for the pooled magnitude of PPFP uptake among postpartum women in Ethiopia, 2024.

We conducted a thorough investigation into the origins of heterogeneity by employing a leave-one-out sensitivity analysis. This analysis demonstrated that the removal of each study from the overall assessment did not significantly impact the estimated average prevalence. The average prevalence consistently fell within the 95% confidence interval of the overall average prevalence calculated when all studies were included. Therefore, no single study exerted a notable influence on the average prevalence. Furthermore, the sensitivity analysis indicated that the exclusion of each study individually yielded an average prevalence of 36.41%, accompanied by a 95% confidence interval ranging from 24.78 to 40.03, as illustrated in Figure 4.

### Meta-regression

Considerable variability was noted among the studies included in the meta-analysis. To investigate the origins of this variability, we performed a meta-regression analysis utilizing sample size, which indicated a significant influence on the observed differences in PPFP uptake, as illustrated in Table 3.

**Table 3:** Meta-regression to identify the source of heterogeneity for the pooled magnitude of PPFP uptake among postpartum women in Ethiopia, 2024.

Variables	*β* (95% CI)	*P*-value
Sample size	.003616 (.0020018, .0052302)	0.000

### Factors associated with postpartum family planning utilization in Ethiopia

This Umbrella of systematic review and meta-analysis identified the most often occurring related variables, which were family planning counseling, couple discussion and maternal education Supplementary file 2.

**Figure 5:**
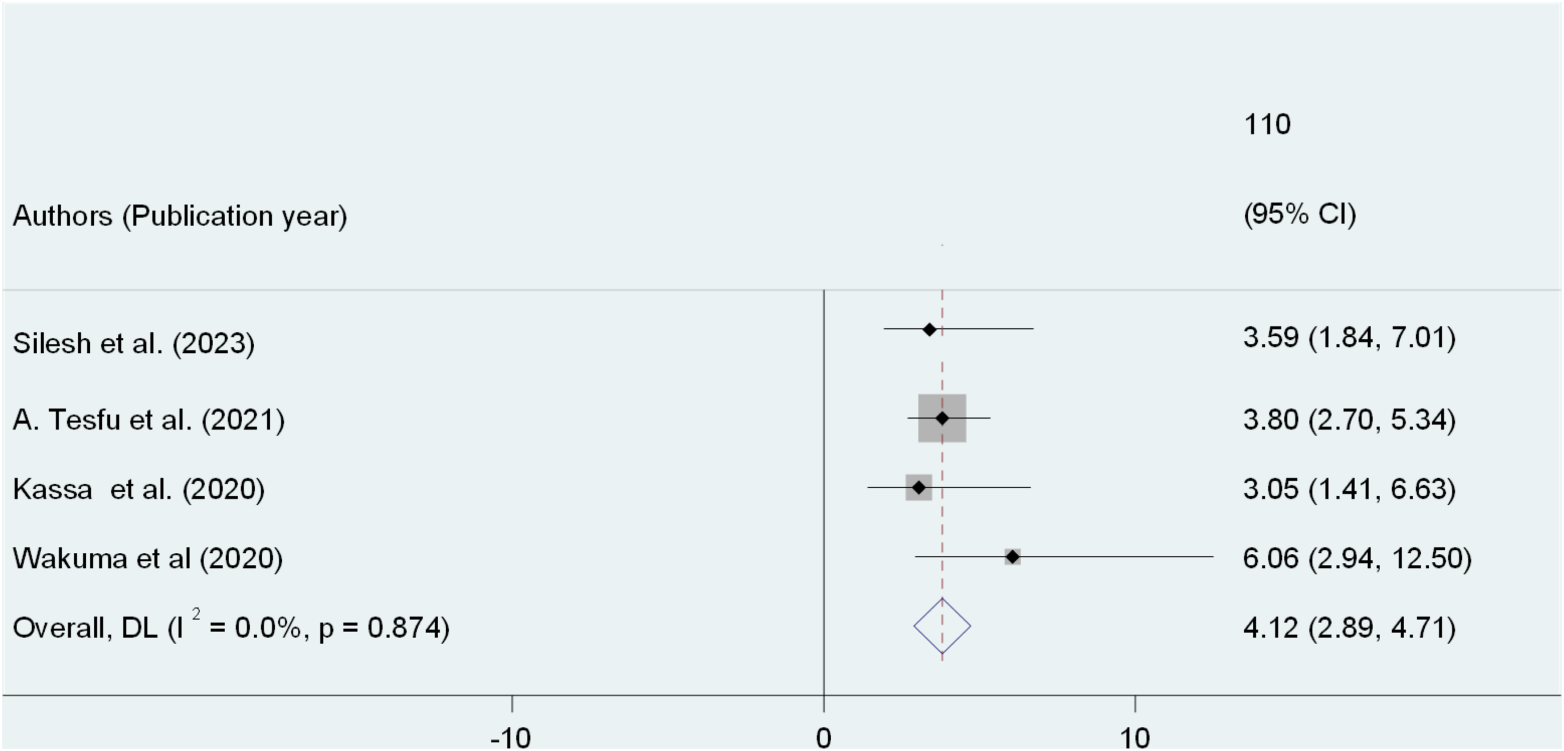
Umbrella review of the association between FP counseling and PPFP use among post-partum women in Ethiopia.

Four SRs included in this umbrella review analyzed family planning counseling (21–24) and the finding revealed that postpartum women who counseled about family planning were 4.12 times more likely to utilize family planning methods than their counterpart (AOR: 4.12, 95% CI: 2.89, 4.71). Moreover, the studies did not reveal any heterogeneity in the results (*I*^2^ = 0.0%, *P* = 0.874) Figure 5.

**Figure 6:**
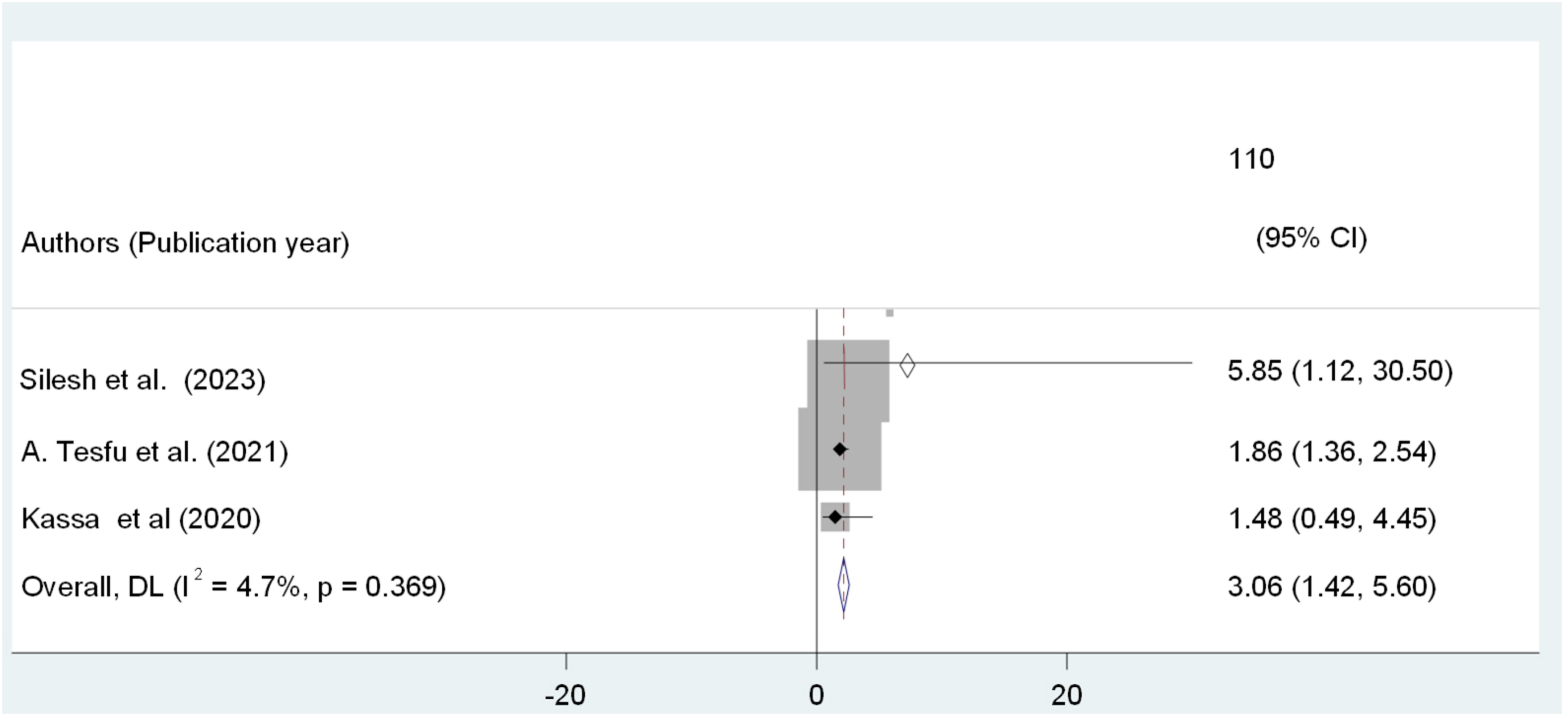
Umbrella review of the association between couples discussion and PPFP use among post-partum women in Ethiopia.

Three out of 5 SRs focused on the couple discussion about family planning during postpartum period (21–23). Postpartum women who had discussed contraception with their partners during the postpartum period were 3.06 times more likely to utilize modern contraceptive methods than their counterparts (AOR: 3.06, 95% CI: 1.42, 5.60). Furthermore, the studies did not showed any heterogeneity in the results (*I*^2^ = 4.7%, *P* = 0.369) Figure 6.

**Figure 7:**
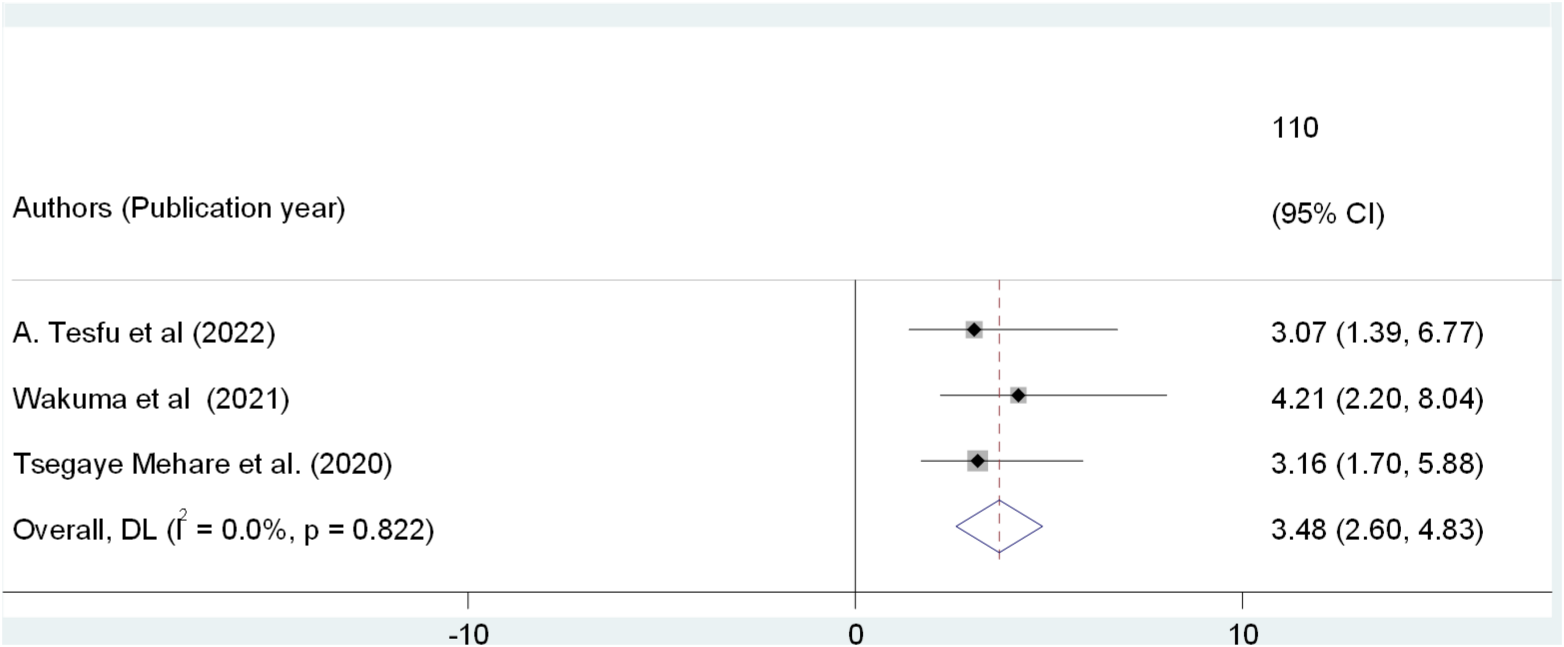
Umbrella review of the association between postnatal follow-up and PPFP use among post-partum women in Ethiopia.

Furthermore, the statistical significance of having post natal follow up among postpartum mothers' regarding PPFP utilization was analyzed using three studies (23–25). Women who had PNC follow up were almost four times more likely to use PPFP methods than those women who had no post natal follow up(AOR: 3.48, 95% CI: 2.60, 4.83). Also, the studies did not showed any heterogeneity in the results (*I*_2_ = 0.0%, *P* = 0.822) Figure 7.

## Discussion

Postpartum family planning (PPFP) plays a crucial role in decreasing high fertility rates among both those who wish to space their children and those who aim to limit family size. It enhances maternal and child health by mitigating the risks associated with unintended short inter-pregnancy intervals and unsafe abortions (2). Currently, approximately 222 million women worldwide experience an unmet need for family planning services (32). Addressing unmet needs in family planning and mitigating the risks associated with closely spaced pregnancies can be achieved through the utilization of postpartum contraceptives (33, 34). Conversely, postpartum women experience amenorrhea for different durations, influenced by their breastfeeding habits. For instance, women who do not engage in breastfeeding may conceive within 45 days following childbirth, while those who do not exclusively breastfeed may also become pregnant before the return of menstruation, leading to a range of pregnancy-related complications (35).

To date, five SRM reports have been published concerning the utilization of PPFP in Ethiopia. These SRM studies are generally regarded as providing robust evidence for decision-making in health programs. Nevertheless, as the volume of individual reviews increases, it may become challenging for individuals seeking information (36). Consequently, this umbrella review was conducted to synthesize the findings from the five SRM studies on PPFP utilization into a comprehensive document. Additionally, several factors, including family planning counseling, couple discussions, and postnatal follow up, were recognized as statistically significant.

The comprehensive review of the five selected systematic review and meta-analysis studies regarding the use of postpartum family planning (PPFP) in Ethiopia produced a summary estimate of 36.41% (95% CI: 24.78, 48.03). This result stands in contrast to findings from studies in Bangladesh at 62.4% (37), Kenya at 86.3% (38), Rwanda at 51.1% (39), Zambia at 45.9% (40), and a systematic review and meta-analysis conducted in low- and middle-income countries, which reported a rate of 41.2% (41). The observed discrepancy may stem from cultural factors, alongside the significant unmet demand for family planning services in Ethiopia (42). Therefore, postpartum family planning (PPFP) services must be provided immediately after childbirth. It's also essential to improve access to basic health facilities in the county and ensure various family planning options, especially PPFP services. Another potential explanation could be the lack of male participation in family planning initiatives within a society where male dominance prevails, as is the case in Ethiopia, where men often wish to have more children than their female partners (43). Additionally, there is a lack of policies that promote male involvement in family planning practices. This includes the absence of support for initiatives aimed at male engagement, social and behavioral change strategies, guidance on collaborative decision-making with partners, and the execution of a holistic approach to male participation in family planning services. Moreover, the discrepancies noted may stem from differences in sample sizes, geographical study locations, and the execution of governmental policies. Our research offers a thorough evaluation of the PPFP across the nation, in contrast to the aforementioned studies, which were confined to specific regions within the country. The findings, however, surpassed the postpartum contraceptive prevalence indicated in the Ethiopian Demographic and Health Survey (EDHS) by 23% (44). This discrepancy may be linked to the EDHS survey's methodology, which covers extensive geographical regions, including hard-to-reach areas. This could result in an underreporting of postpartum family planning (PPFP) usage, particularly among postpartum women living in rural locations with a history of home deliveries, who may lack access to maternal health services. In addition to this, the results observed exceeded the rates documented in studies carried out in Ethiopia, which reported a figure of 21.04% (21). This variation may be attributed to factors such as the previously mentioned finding being derived from a single systematic review and meta-analysis (SRMA) that specifically concentrated on immediate postpartum family planning utilization among postpartum women in Ethiopia. Conversely, the current study encompassed five SRMA studies and addressed both immediate and extended postpartum family planning uptake among postpartum mothers.

The umbrella review revealed that postpartum women who engaged in discussions about contraception with their partners during the postpartum period were 3.06 times more likely to adopt modern contraceptive methods compared to those who did not. This observation is corroborated by findings from other studies conducted in Nigeria (45) and Congo (46).

Ethiopia has been actively pursuing various long-term strategies aimed at transforming its health sector. A significant objective within these strategies is to decrease the unmet need for family planning (FP) from 22% to 10%, which has been recognized as a critical impact indicator in the national health policy. Engaging couples in discussions regarding the utilization of family planning services may play a pivotal role in achieving this goal. Therefore, the government needs to integrate male participation into reproductive health policies. This approach would facilitate communication and interaction between partners, enabling them to acquire essential information about family planning and make informed decisions regarding the use of maternal health services. Since family planning is a shared responsibility, enhancing this dialogue could lead to an increased intention to utilize contraception following childbirth.

This umbrella review revealed that postpartum women who received counseling on family planning were 4.12 times more likely to adopt family planning methods compared to those who did not receive such counseling. This conclusion is corroborated by research conducted in India, Nepal, Sri Lanka, and Tanzania (47). Women who participate in family planning counseling may gain a more comprehensive understanding of the various family planning methods, including their advantages and disadvantages. This increased awareness of birth spacing through contraceptive use following childbirth can improve their decision-making abilities regarding postpartum family planning and encourage them to utilize contraceptives.

The analysis indicated that women who participated in postnatal care (PNC) follow-up were nearly four times more inclined to adopt postpartum family planning (PPFP) methods compared to those who did not engage in postnatal follow-up. This observation is corroborated by research conducted in Kenya and Zambia (40). It is suggested that women attending PNC appointments during the postpartum phase likely receive comprehensive guidance on the significance of postpartum family planning. Consequently, they may exhibit a greater motivation to implement the methods they choose.

This umbrella review of systematic reviews and meta-analyses exhibits significant strengths, such as the application of varied search strategies, a thorough evaluation of methodological quality, compliance with the PRISMA 2020 extension guidelines, and the execution of a funnel test. To our knowledge, no extensive assessment in the form of an umbrella review has been performed regarding postpartum family planning utilization in Ethiopia, despite the existence of numerous empirical studies and specific systematic reviews and meta-analyses. However, this review is not without its limitations; it exclusively includes articles published in English which could introduce a potential bias by excluding studies published in other languages and is constrained by a limited number of studies. Additionally, despite significant attempts to tackle the issue, heterogeneity remained evident among the studies included, suggesting that there were discrepancies in methodologies or populations that were not entirely resolved. One additional limitation is that one of the systematic reviews included women of reproductive age, pregnant women, and postpartum women (24), which may have introduced biases. The substantial variation in sample sizes across studies (ranging from 4,367 to 11,932) and the considerable variation in prevalence, lack of analysis regarding regional variations within Ethiopia, and the absence of analysis regarding specific contraceptive methods preferred and cost-effectiveness considerations limits the practical utility of the findings were additional limitations. Moreover, the absence of comparable reviews from other countries further hampers our ability to reach significant conclusions, as these were primary studies. To overcome these limitations and enhance the depth of future research, it is recommended to adopt a more inclusive methodology. In particular, the integration of interventional studies into the research framework is suggested, as this could significantly strengthen the overall validity of the findings and lead to a more thorough and nuanced comprehension of the topic at hand.

## Conclusion

The overall prevalence of postpartum family planning (PPFP) was determined to be 36.41%, highlighting a significant gap that requires attention. Contributing factors to this situation include the availability of family planning counseling, discussions between couples, and postnatal follow-up care. These results emphasize the necessity for focused interventions aimed at increasing the utilization of PPFP services, as well as improving postnatal follow-up, family planning counseling, and couple discussions. Consequently, our findings strongly recommend that special consideration be given to mothers. Furthermore, policymakers in the health sector, along with promoters, non-governmental organizations, community organizations, and other relevant stakeholders, should initiate educational programs on family planning that emphasize the health advantages of postpartum contraceptive use, particularly in preventing unintended pregnancies and prolonging inter-pregnancy intervals. Healthcare providers should advocate for breastfeeding and introduce the lactational amenorrhea method (LAM) alongside other immediate postpartum contraceptive options. It is also essential for contraceptive programs to involve men in the promotion and uptake of family planning services.

## Implication of the review

This research provides current and succinct evidence regarding the uptake of postpartum family planning (PPFP) in Ethiopia. It serves as a valuable resource for program developers and implementers across various sectors, including government, non-governmental organizations, bilateral and multilateral agencies, the private sector, as well as charitable and civic institutions that aim to deliver standardized family planning services in Ethiopia. The findings underscore the importance of preventing closely spaced pregnancies, which allows families to better support their children, invest in their education, enhance child health, and enable women at risk of pregnancy-related complications to space and delay pregnancies. Furthermore, the study highlights the essential role of male involvement, both from a programmatic perspective and as a means to achieve gender equity in reproductive rights and responsibilities. It stresses the necessity of providing health education and family planning counseling services that guarantee full, free, and informed choices while maintaining privacy and confidentiality, which are critical for ensuring the quality of family planning services. Additionally, this study identifies key determinants influencing the uptake of PPFP and proposes strategies to address these issues in Ethiopia, such as enhancing family planning counseling, encouraging couple discussions, and ensuring postnatal follow-up, particularly by promoting male participation in family planning to improve communication between couples regarding fertility and family planning, thereby ensuring that decisions reflect the needs and preferences of both partners.

## Data Availability

The original contributions presented in the study are included in the article/Supplementary Material, further inquiries can be directed to the corresponding author.
