# Supplementary material for: Magnitude and associated factors of postpartum family planning uptake among postpartum women in Ethiopia: an umbrella review
Source: Front Glob Womens Health. 2024 Dec 18;5:1481601. doi: 10.3389/fgwh.2024.1481601 (PMC11697147; doi:10.3389/fgwh.2024.1481601)
Supplement: Supplementary file 1 [file Table1.docx]

**Supplemental S1 File – Search Strategy Summary**

The following search terms were used across all English language databases with minor adaptations:

| Period search was conducted | June 15- July 15, 2024 |
| --- | --- |
| Inclusion  criteria | - Systematic reviews and meta-analyses , - Studies published until July 15, 2024 - Studies conducted in Ethiopia. - Postpartum women - All published studies in the English - Studies reported the magnitude of modern postpartum contraceptive use |
| Exclusion  criteria | - Case reports - case series - letters to editors - Conference papers or book chapters - Studies lacked a clear research question, search strategy, or defined process for selecting article |
| Libraries | Worldwide |
| Records identified from secondary databases, google scholar | The search terms were combined using Boolean operators "OR" and "AND". For the online database, the following mesh terms or keywords were utilized: postpartum OR post-delivery OR parturition OR puerperium OR immediate postpartum OR extended postpartum AND prevalence OR magnitude OR proportion AND use OR utilization OR intention OR unmet need OR barrier AND Predictors OR contraception, OR contraceptive, OR family planning, OR modern contraceptives, OR modern postpartum family planning, OR modern family planning, AND Ethiopia AND Systematic review |
