# Supplementary material for: Magnitude and associated factors of postpartum family planning uptake among postpartum women in Ethiopia: an umbrella review
Source: Front Glob Womens Health. 2024 Dec 18;5:1481601. doi: 10.3389/fgwh.2024.1481601 (PMC11697147; doi:10.3389/fgwh.2024.1481601)
Supplement: Supplementary file 2 [file Table2.docx]

**Supplemental file 2 Meta-analysis of pooled associated factors of postpartum family planning use in Ethiopia, 2024**

| Variable | OR(95% CI) | Heterogeneity | Number of review included |
| --- | --- | --- | --- |
| Family planning counseling | 4.12 (2.89, 4.71) | I^2^=0.0%, P=0.87 | 4 |
| Couple discussion | 3.06 (1.42, 5.60) | I^2^=4.7%, P=0.369 | 3 |
| Post natal follow up | 3.93 (2.84,4.92) | I^2^=0.0%, P=0.605 | 3 |
